# Expanding the Chemical Information Science gateway

**DOI:** 10.12688/f1000research.52192.1

**Published:** 2021-04-16

**Authors:** José L. Medina-Franco

**Affiliations:** 1 DIFACQUIM research group, Department of Pharmacy, School of Chemistry, Universidad Nacional Autónoma de México, Mexico City 04510, Mexico

**Keywords:** Chemical Information science, chemoinformatics, bioinformatics, informatics, machine learning, metabolomics, open science

## Abstract

As chemical information evolves, impacting many chemistry areas, effective ways to disseminate results by the scientific community are also changing. Thus, publication schemes adapt to meet the needs of researchers across disciplines to share high-quality data, information, and knowledge. Since 2015, the F1000Research Chemical Information Science (CIS) gateway has offered an open and unique model to disseminate science at the interface of chemoinformatics, bioinformatics, and several other informatic-related disciplines. In response to the evolution of chemical information science, the F1000Research CIS gateway has incorporated new members to the advisory board. It is also reinforcing and expanding the gateway areas with a particular focus on machine learning and metabolomics. The range of available article types, availability of data, exposure within complementary multidisciplinary F1000Research gateways, and indexing in major bibliographic databases increases the visibility of all contributions. As part of progressing open science in this field, we look forward to your high-quality contributions to the CIS gateway.

## A new era of chemical information

Since the previous update of the F1000Reseach Chemical Information Science (CIS) gateway, ^1^ the overarching goals and applications of chemical information have evolved significantly with deeper and broader applications in a large number of areas including, but not limited to drug discovery, material sciences, metabolomics and natural product chemistry, organic, analytical, and food chemistry, to name a few. ^2^^,^^3^ In the new era of chemical information, we are delighted to welcome some new members to our advisory board, including several early career researchers across different disciplines within chemical information science and representing a diverse range of geographical regions. These new advisors will help to deepen the range of scientific interests, backgrounds, and experiences which are shaping this exciting body of research, as well as ultimately widening the gateway’s audience to further increase the dissemination of the work within it.

## A unique publication model

The F1000Research publishing model is designed to speed up the sharing of research, improve transparency and reproducibility, and reduce editorial barriers to publishing. Articles are published rapidly as soon as they are accepted after passing a series of rigorous but expedited prepublication checks to assess originality, readability, availability of FAIR data, and compliance with policies and ethical guidelines. This rapid dissemination of research removes the delay in others’ ability to benefit from accessing the work during the reviewing period. Peer review by independent invited experts, suggested by the authors, takes place openly after publication. Transparent peer review helps to address potential biases in the process, adds value to the article in question, provides reviewers with credit for their work, and can provide better written and more constructive feedback than anonymous peer review, as shown in randomized clinical trials. ^4^ Authors are encouraged to respond openly to the peer review reports, which are published with the article, and can publish revised versions of their article at no further cost, if they wish. An article remains published regardless of the reviewers’ reports and findable via Google Scholar. Articles that pass peer review are then indexed in Scopus (receiving a Citation Score), PubMed, Chemical Abstracts Service, and other bibliographic databases. The advantages of the F1000Research publishing model have been highlighted by the COVID-19 pandemic which has shown the increasing importance of data availability and rapid dissemination of research. In chemical information science, for example, the drug discovery process can be accelerated by the early sharing of results, allowing others access to the work while expert reviewers assess it. This contrasts with the closed review process in traditional journals, which can take several months to complete. In chemical information, it is frequently desirable to share, for instance, full data, open-source code, or free servers that can have a significant impact in the community and represent advances in the field. Readers can also benefit from the reviewers’ comments and opinions that are valuable in many cases but are lost or hidden from the public in traditional publishing; professionals, educators, and students will also certainly benefit from reading the open peer review and discussion of the contributions. Furthermore, since chemical information is moving so rapidly, as illustrated by the impressive advancements within, for instance, artificial intelligence, ^5^^,^^6^ waiting several months to publish the paper after peer review can jeopardize the research impact and novelty.

## A broad range of manuscript types

A key objective for the CIS gateway moving forward will be to encourage a broad range of contributions that benefit researchers and add value to the community. These can be in the form of original research and review articles but also includes articles for which authors may have struggled to find a ‘home’ for via other traditional journal publication venues. The publication of null or negative results is one such example; for instance, studies showing why a computational method was not capable of solving a particular problem. This approach will allow researchers credit for a wider range of the outputs they are producing, reduce research waste, and bring increased visibility of valuable information for the community. Furthermore, the wide range of article types offered by F1000Research, such as Data Notes, Software Tools, and Method Articles, will also be important as we advance the gateway. These article types provide an ideal format for the dissemination of research specific to chemical information science, and we are eager to see researchers in the field take advantage. Researchers can present new or updated open-source, free applications, or implementations in web servers, for example. Readers that include expert practitioners or newcomers to the chemical information field, students, and professionals in related areas will also be able to keep up to date with developments and applications in chemical information science. Article types such as Data Notes, Software Tools, and Methods will disseminate useful resources that can be put into practice immediately in research projects.

## Supporting the interdisciplinary nature of research

Since the initial launch of the CIS gateway, several other gateways and collections have been created on F1000Research, which complement the subject matter and methodologies used within chemical information science. For instance, the Python collection was an ideal candidate for coordination with the CIS gateway, and in 2020 we launched a successful joint call for papers which resulted in the publication of several Software Tool articles. A range of other gateways, such as RPackage, Bioconductor, and the International Society for Computational Biology Community Journal, provide excellent opportunities for collaboration in the future. The ability to publish across different gateways and collections is an offering unique to F1000Research. These articles benefit from additional visibility in the form of increased views, downloads, and potentially, citations. Sharing materials and data across communities also acknowledges the interdisciplinary nature of research and presents exciting opportunities for future collaboration.

## Reinforcing and expanding the gateway areas

In addition to the broad applications of chemical information in research areas such as organic, analytical, food, and natural products chemistry, as well as material and environmental sciences, ^2^^,^^3^^,^^7^^,^^8^ we are now pleased to announce the launch of two new gateway areas. These new areas will focus on machine learning and metabolomics, aiming to contribute to the broader literature while also exploring the current application areas. Machine learning is now reshaping the development and applications of chemical information in inter- and multi-disciplinary areas. ^9^ Clear examples are, but are not limited to, drug discovery and environmental chemistry, both of which have a significant impact on human health. ^10^ Similarly, chemical information can make a significant contribution to advances in metabolomics. ^11^ We look forward to your submissions to the F1000Research CIS gateway to further advance the chemical information sciences.

## Data availability

No data are associated with this study.
